# Supplementary material for: A FRET-based study reveals site-specific regulation of spindle position checkpoint proteins at yeast centrosomes
Source: eLife. 2016 May 9;5:e14029. doi: 10.7554/eLife.14029 (PMC4878874; doi:10.7554/eLife.14029)
Supplement: Supplementary file 1. — Descriptions of the yeast strains and plasmids used in this study are listed in this file. DOI: http://dx.doi.org/10.7554/eLife.14029.040 [file elife-14029-supp1.docx]

**Supplemental File 1**

**Table of Yeast Strains and Plasmids**

| **Strains** | **Description** | **Source / Reference** |
| --- | --- | --- |
| YGY236 | ESM356-1 *BFA1-EYFP-mTUR-hphNT1* | this study |
| YGY044 | ESM356-1 *BFA1-mTUR-hphNT1* | this study |
| YGY099 | ESM356-1 *NUD1-mTUR-hphNT1 BFA1-EYFP-HIS3MX6* | this study |
| YGY084 | ESM356-1 *NUD1-mTUR-hphNT1* | this study |
| YGY097 | ESM356-1 *SPC72-mTUR-hphNT1 BFA1-EYFP-HIS3MX6* | this study |
| YGY083 | ESM356-1 *SPC72-mTUR-hphNT1* | this study |
| YGY229 | ESM356-1 *CNM67-mTUR-hphNT1 BFA1-EYFP-HIS3MX6* | this study |
| YGY051 | ESM356-1 *CNM67-mTUR-hphNT1* | this study |
| YGY222 | ESM356-1 *natNT2-*Gal1*-mTUR-CNM67* | this study |
| YGY111 | ESM356-1 Gal1*-CDC20-LEU2 NUD1-mTUR-hphNT1 BFA1-EYFP-HIS3MX6* | this study |
| YGY108 | ESM356-1 Gal1*-CDC20-LEU2 NUD1-mTUR-hphNT1* | this study |
| YGY109 | ESM356-1 Gal1*-CDC20-LEU2 SPC72-mTUR-hphNT1 BFA1-EYFP-HIS3MX6* | this study |
| YGY107 | ESM356-1 Gal1*-CDC20-LEU2 SPC72-mTUR-hphNT1* | this study |
| YGY078 | ESM356-1 Gal1*-CDC20-LEU2* | this study |
| YGY105 | ESM356-1 *ura3-52::*Gal1*-clb2ΔDB-URA3 NUD1-mTUR-hphNT1 BFA1-EYFP-HIS3MX6* | this study |
| YGY102 | ESM356-1 *ura3-52::*Gal1*-clb2ΔDB-URA3 NUD1-mTUR-hphNT1* | this study |
| YGY103 | ESM356-1 *ura3-52::*Gal1*-clb2ΔDB-URA3 SPC72-mTUR-hphNT1 BFA1-EYFP-HIS3MX6* | this study |
| YGY101 | ESM356-1 *ura3-52::*Gal1*-clb2ΔDB-URA3 SPC72-mTUR-hphNT1* | this study |
| YGY077 | ESM356-1 *ura3-52::*Gal1*-clb2ΔDB-URA3* | this study |
| YGY128 | ESM356-1 Gal1*-KIN4-kanMX6 NUD1-mTUR-hphNT1 BFA1-EYFP-HIS3MX6* | this study |
| YGY127 | ESM356-1 Gal1*-KIN4-kanMX6 NUD1-mTUR-hphNT1* | this study |
| YGY204 | ESM356-1 Gal1*-KIN4-kanMX6 SPC72-mTUR-hphNT1 BFA1-EYFP-HIS3MX6* | this study |
| YGY202 | ESM356-1 Gal1*-KIN4-kanMX6 SPC72-mTUR-hphNT1* | this study |
| DLY90 | ESM356-1 Gal1*-KIN4-kanMX6* | this study |
| YGY210 | ESM356-1 *bmh1Δ::klTRP1* Gal1*-KIN4-kanMX6 NUD1-mTUR-hphNT1 BFA1-EYFP-HIS3MX6* | this study |
| YGY208 | ESM356-1 *bmh1Δ::klTRP1* Gal1*-KIN4-kanMX6 NUD1-mTUR-hphNT1* | this study |
| YGY211 | ESM356-1 *bmh1Δ::klTRP1* Gal1*-KIN4-kanMX6 SPC72-mTUR-hphNT1 BFA1-EYFP-HIS3MX6* | this study |
| YGY209 | ESM356-1 *bmh1Δ::klTRP1* Gal1*-KIN4-kanMX6 SPC72-mTUR-hphNT1* | this study |
| YGY207 | ESM356-1 *bmh1Δ::klTRP1* Gal1*-KIN4-kanMX6* | this study |
| YGY215 | ESM356-1 *bmh1Δ::klTRP1 NUD1-mTUR-hphNT1 BFA1-EYFP-HIS3MX6* | this study |
| YGY213 | ESM356-1 *bmh1Δ::klTRP1 NUD1-mTUR-hphNT1* | this study |
| YGY214 | ESM356-1 *bmh1Δ::klTRP1 SPC72-mTUR-hphNT1 BFA1-EYFP-HIS3MX6* | this study |
| YGY212 | ESM356-1 *bmh1Δ::klTRP1 SPC72-mTUR-hphNT1* | this study |
| YMY392 | ESM356-1 *bmh1Δ::klTRP1* | Caydasi *et al*., 2014 |
| YGY228 | ESM356-1 *kar9Δ::klTRP1 NUD1-mTUR-hphNT1 BFA1-EYFP-kanMX6* | this study |
| YGY230 | ESM356-1 *kar9Δ::klTRP1 NUD1-mTUR-hphNT1* | this study |
| YGY237 | ESM356-1 *kar9Δ::klTRP1 SPC72-mTUR-hphNT1 BFA1-EYFP-kanMX6* | this study |
| YGY238 | ESM356-1 *kar9Δ::klTRP1 SPC72-mTUR-hphNT1* | this study |
| BKY035 | ESM356-1 *kar9Δ::HIS3MX6* | this study |
| YGY263 | BMA64 *spc72Δ::kanMX6 NUD1-mTUR-hphNT1 BFA1-EYFP-HIS3MX6* | this study |
| YGY262 | BMA64 *spc72Δ::kanMX6 NUD1-mTUR-hphNT1* | this study |
| YGY261 | BMA64 *spc72Δ::kanMX6* | this study |
| YGY302 | BMA64 *leu2Δ1::LEU2*-*mCherry-TUB1BFA1-GFP-klTRP1 kar9Δ::kanMX6* | this study |
| YGY303 | BMA64 *leu2Δ1::LEU2*-*mCherry-TUB1 BFA1-GFP-klTRP1 kar9Δ::kanMX6 kin4Δ::hphNT1* | this study |
| YGY310 | BMA64 *leu2Δ1::LEU2*-*mCherry-TUB1 BFA1-GFP-klTRP1 spc72Δ::kanMX6* | this study |
| AKY2104 | BMA64 *BFA1-GFP-klTRP1 kar9Δ::kanMX6* | this study |
| YGY266 | BMA64 *BFA1-GFP-klTRP1 kar9Δ::kanMX6 kin4Δ::hphNT1* | this study |
| AKY2182 | BMA64 *BFA1-GFP-klTRP1 spc72Δ::kanMX6* | this study |
| YGY314 | BMA64 *LEU2-BFA1(2A)-GFP-hphNT1::bfa1Δ::klTRP1* | this study |
| YGY290 | BMA64 *KIN4-GFP-hphNT1 SPC42-eqFP-HIS3MX6* | this study |
| YGY291 | BMA64 *KIN4-GFP-hphNT1 SPC42-eqFP-HIS3MX6 spc72∆::kanMX6* pRS316*-SPC72* | this study |
| YGY292 | BMA64 *KIN4-GFP-hphNT1 SPC42-eqFP-HIS3MX6 spc72∆::kanMX6* pRS316*-SPC72* | this study |
| YGY288 | BMA64 *ade2-1::ADE2-GFP-TUB1 kar9∆::HIS3MX6* | this study |
| YGY289 | BMA64 *ade2-1::ADE2-GFP-TUB1 kar9∆::HIS3MX6 kin4∆::klTRP1* | this study |
| YGY287 | BMA64 *ade2-1::ADE2-GFP-TUB1 spc72∆::kanMX6* | this study |
| YGY047 | ESM356-1 *SPC42-mTUR-hphNT1* | this study |
| YGY046 | ESM356-1 *SPC42-mTUR-hphNT1 CNM67-EYFP-HIS3MX6* | this study |
| YGY043 | ESM356-1 *SPC110-mTUR-hphNT1* | this study |
| YGY053 | ESM356-1 *SPC110-mTUR-hphNT1 CNM67-EYFP-HIS3MX6* | this study |
| KCY2 | YPH499 *nud1∆::kanMX6 leu2Δ1::LEU2*-*nud1-2(ts)* | Gruneberg *et al.*, 2000 |
| YMK205 | YPH499 *spc72-7(ts)* | Knop and Schiebel, 1998 |
| ESM432-2 | YPH499 *cnm67∆::HIS3MX6* pRS316-*CNM67* | this study |
| YGY192 | ESM356-1 *natNT2-*Gal1*-mTUR-BUB2* | this study |
| YGY198 | ESM356-1 *natNT2-*Gal1*-mTUR-BUB2 BFA1-EYFP-HIS3MX6* | this study |
| YGY200 | ESM356-1 *natNT2-*Gal1*-mTUR-BUB2 NUD1-EYFP-HIS3MX6* | this study |
| YGY199 | ESM356-1 *natNT2-*Gal1*-mTUR-BUB2 SPC72-EYFP-HIS3MX6* | this study |
| YGY347 | ESM356-1 *kar9Δ::klTRP1 kin4Δ::hphNT2 NUD1-mTUR-hphNT1 BFA1-EYFP-kanMX6* | this study |
| YGY351 | ESM356-1 *kar9Δ::klTRP1 kin4Δ::hphNT2 NUD1-mTUR-hphNT1* | this study |
| YGY352 | ESM356-1 *kar9Δ::klTRP1 kin4Δ::hphNT2 SPC72-mTUR-hphNT1 BFA1-EYFP-kanMX6* | this study |
| YGY227 | ESM356-1 *kar9Δ::klTRP1 kin4Δ::hphNT2 SPC72-mTUR-hphNT1* | this study |
| YGY348 | ESM356-1 *kar9Δ::klTRP1 kin4Δ::hphNT2* | this study |
| YGY157 | ESM356-1 *kar9∆::natNT2 BFA1-mTUR-hphNT1* | this study |
| YGY219 | ESM356-1 *kar9∆::hphNT1 BFA1-EYFP-HIS3MX6* | this study |
| YGY317 | ESM356-1 *kar9∆::HIS3MX6 natNT2-*Gal1*-EYFP-BFA1* | this study |
| YGY340 | ESM356-1 *kar9∆::HIS3MX6 natNT2-*Gal1*-mTUR-BFA1* | this study |
| AKY263 | YPH499 *kar9∆::klTRP1 bfa1∆::HIS3MX6* | this study |
| AKY269 | YPH499 *kar9∆::klTRP1 bub2∆::HIS3MX6* | this study |
| YGY151 | ESM356-1 *kar9∆::HIS3MX6 BUB2-mTUR-hphNT1* | this study |
| YGY147 | ESM356-1 *kar9∆::HIS3MX6 BUB2-EYFP-kanMX6* | this study |
| YGY194 | YPH499 *kar9∆::HIS3MX6 natNT2-*Gal1*-mTUR-BUB2* | this study |
| YGY158 | ESM356-1 *kar9∆::natNT2 BFA1-mTUR-hphNT1 BUB2-EYFP-kanMX6* | this study |
| YGY149 | ESM356-1 *kar9∆::HIS3MX6 BUB2-GFP-klTRP1* | this study |
| YGY293 | ESM356-1 *SPC72-mTUR-hphNT1 natNT2-*Gal1*-EYFP-BFA1* | this study |
| YGY294 | ESM356-1 *NUD1-mTUR-hphNT1 natNT2-*Gal1*-EYFP-BFA1* | this study |
| YGY199 | ESM356-1 *SPC72-EYFP-kanMX6 natNT2-*Gal1*-mTUR-BUB2* | this study |
| YGY200 | ESM356-1 *NUD1-EYFP-kanMX6 natNT2-*Gal1*-mTUR-BUB2* | this study |
| YGY217 | ESM356-1 *kar9∆::klTRP1* pGW399 | this study |
| AKY315 | ESM356-1 *kar9∆::HIS3MX6* pGW399 *bfa1∆::klTRP1* | this study |
| YGY220 | ESM356-1 *kar9∆::klTRP1* pGW399 *SPC72-mTUR- hphNT1 BFA1-EYFP-kanMX6* | this study |
| YGY221 | ESM356-1 *kar9∆::klTRP1* pGW399 *NUD1-mTUR- hphNT1 BFA1-EYFP-kanMX6* | this study |
| AKY260 | ESM356-1 *kar9∆::HIS3MX6* pGW399 | this study |
| AKY2876 | ESM356-1 *kar9∆::HIS3MX6* pGW399 *SPC72-GBP-kanMX6* | this study |
| AKY2878 | ESM356-1 *kar9∆::HIS3MX6* pGW399 *SPC72-GBP-kanMX6 BFA1-GFP-klTRP1* | this study |
| AKY2844 | ESM356-1 *kar9∆::HIS3MX6* pGW399 *BFA1-GFP-klTRP1* | this study |
| AKY321 | ESM356-1 *kar9∆::HIS3MX6* pGW399 *kin4∆::klTRP1* | this study |
| AKY2895 | ESM356-1 *kar9∆::HIS3MX6* pGW399 *BFA1-GFP-klTRP1 kin4∆::hphNT1* | this study |
| AKY2897 | ESM356-1 *kar9∆::HIS3MX6* pGW399 *SPC72-GBP-kanMX6 BFA1-GFP-klTRP1 kin4∆::hphNT1* | this study |
| AKY2898 | ESM356-1 *kar9∆::HIS3MX6* pGW399 *BFA1-GFP-klTRP1 natNT2*-Gal1-*CDC5* | this study |
| AKY2900 | ESM356-1 *kar9∆::HIS3MX6* pGW399 *SPC72-GBP-kanMX6 BFA1-GFP-klTRP1 natNT2*-Gal1-*CDC5.* | this study |
| YPH499 | *MATa ura3-52 lys2-801amber ade2-101ochre trp1∆63 his3∆200 leu2∆1* | Siroski and Hieter, 1989 |
| BMA64 | *MATa ura3-1 trp1-∆2 leu2-3, 112 his3-11 ade2-1 can1-100* | Baudin-Bailieu *et al*., 1997 |
| ESM356-1 | *MATa ura3-52 leu2Δ1 his3Δ200 trp1Δ63* | Pereira *et al*., 2001 |
|  |  |  |
| **Plasmids** | **Description** | **Source / Reference** |
| pSM447 | pRS305-*SPC72* | Knop and Schiebel, 1998 |
| pGW399 | pRS316-*KAR9* | Caydasi *et al*., 2010 |
| pAK11 | pRS306-*mCherry-TUB1* | Khmelinskii *et al.*, 2007 |
| pSM1027 | pRS402-*GFP-TUB1* | Caydasi *et al.*, 2010 |
| pUJ011 | p426-Met25-*KIN4* | Caydasi *et al*., 2010 |
| c2434-1 | YIplac211-Gal1**-***clb2ΔDB* | Surana *et al*., 1993 |
| pYM-*mTUR* | pYM-*mTUR-*hphNT1 gift from M. Knop (ZMBH, University of Heidelberg, Germany) | |
| pYG1 | pYM-*EYFP-mTUR* fusion | this study |
| pYG2 | pYM-Gal1-*mTUR* | this study |
| pYG3 | pYM-Gal1-EYFP | this study |
| MBP | pMal-2c-*MBP* | Maekawa *et al*., 2007 |
| MBP-*BFA1* | pMal-2c-*MBP-BFA1* | Maekawa *et al*., 2007 |
| pSM413 | pGEX-4T-1-*GST*-*SPC72* | Maekawa *et al*., 2007 |
| pMK297 | pET-28c-*6His*-*spc72^231-622^ (spc72-C)* | this study |
| pMF523 | pET-28c-*6His*-*MLC1* | this study |
